# Supplementary material for: MED16 Promotes Tumour Progression and Tamoxifen Sensitivity by Modulating Autophagy through the mTOR Signalling Pathway in ER-Positive Breast Cancer
Source: Life (Basel). 2022 Sep 20;12(10):1461. doi: 10.3390/life12101461 (PMC9604881; doi:10.3390/life12101461)
Supplement: Supplementary file 1 [file life-12-01461-s001.zip › life-1874856-supplementary/Supplement Table/Supplement Table1.pdf]

**R**			**Risk ratio (RR)**		**Statistical tests**					
Process groups	Reference groups	HR	confidence interval (95%CI)		Likelihood ratio test		Wald test		logrank test	
			Lower	Upper	W value	P value	W value	P value	W value	P value
**H**	L	4.13	2.84	5.99	51.03	9.1e-13	55.31	1.0e-13	64.87	8.0e-16
