# Supplementary figures and images for: MED16 Promotes Tumour Progression and Tamoxifen Sensitivity by Modulating Autophagy through the mTOR Signalling Pathway in ER-Positive Breast Cancer
Source: Life (Basel). 2022 Sep 20;12(10):1461. doi: 10.3390/life12101461 (PMC9604881; doi:10.3390/life12101461)

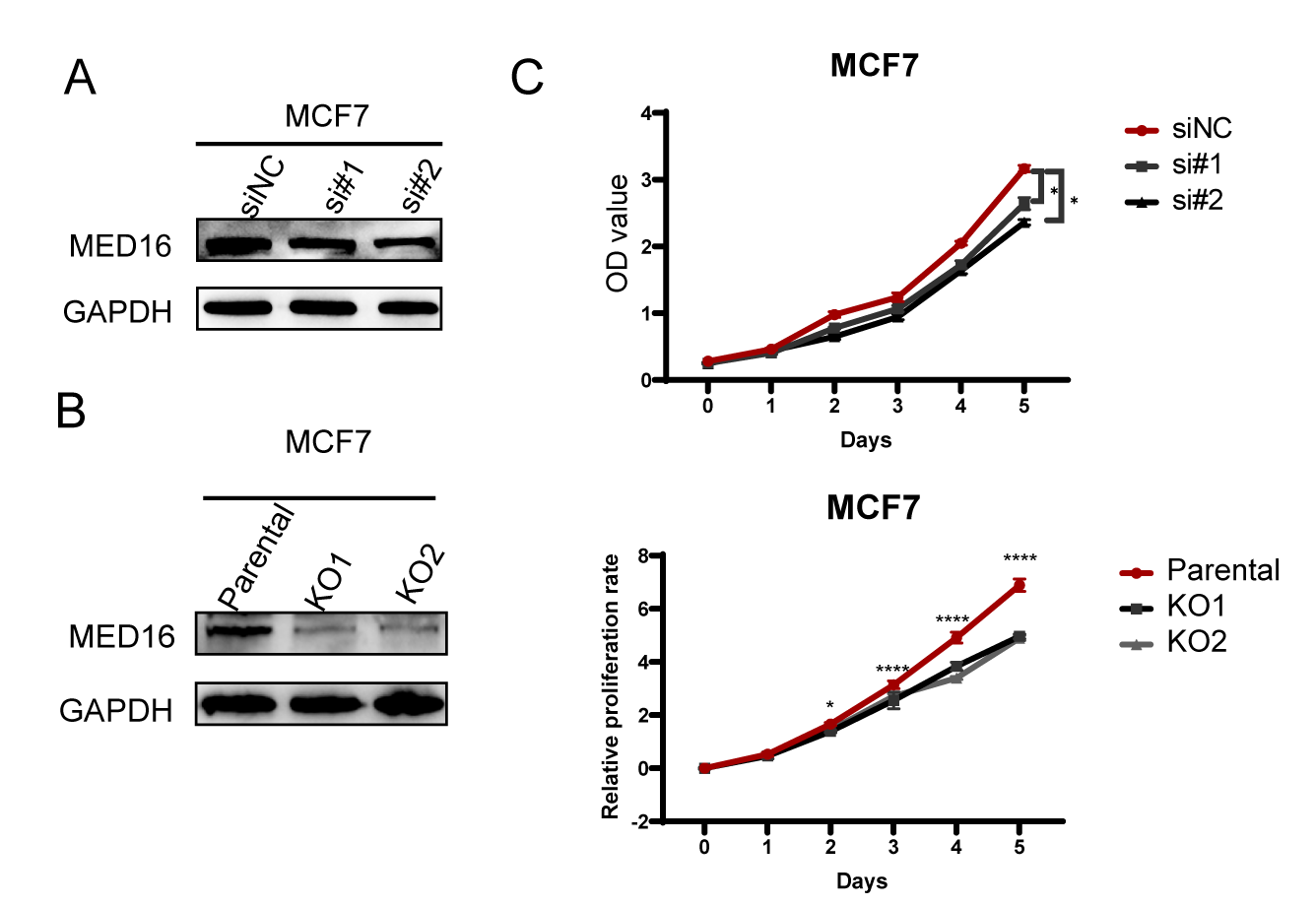

Supplement: Supplementary file 1 [file life-12-01461-s001.zip › life-1874856-supplementary/Supplement Figure/Supplement figure1.tif]

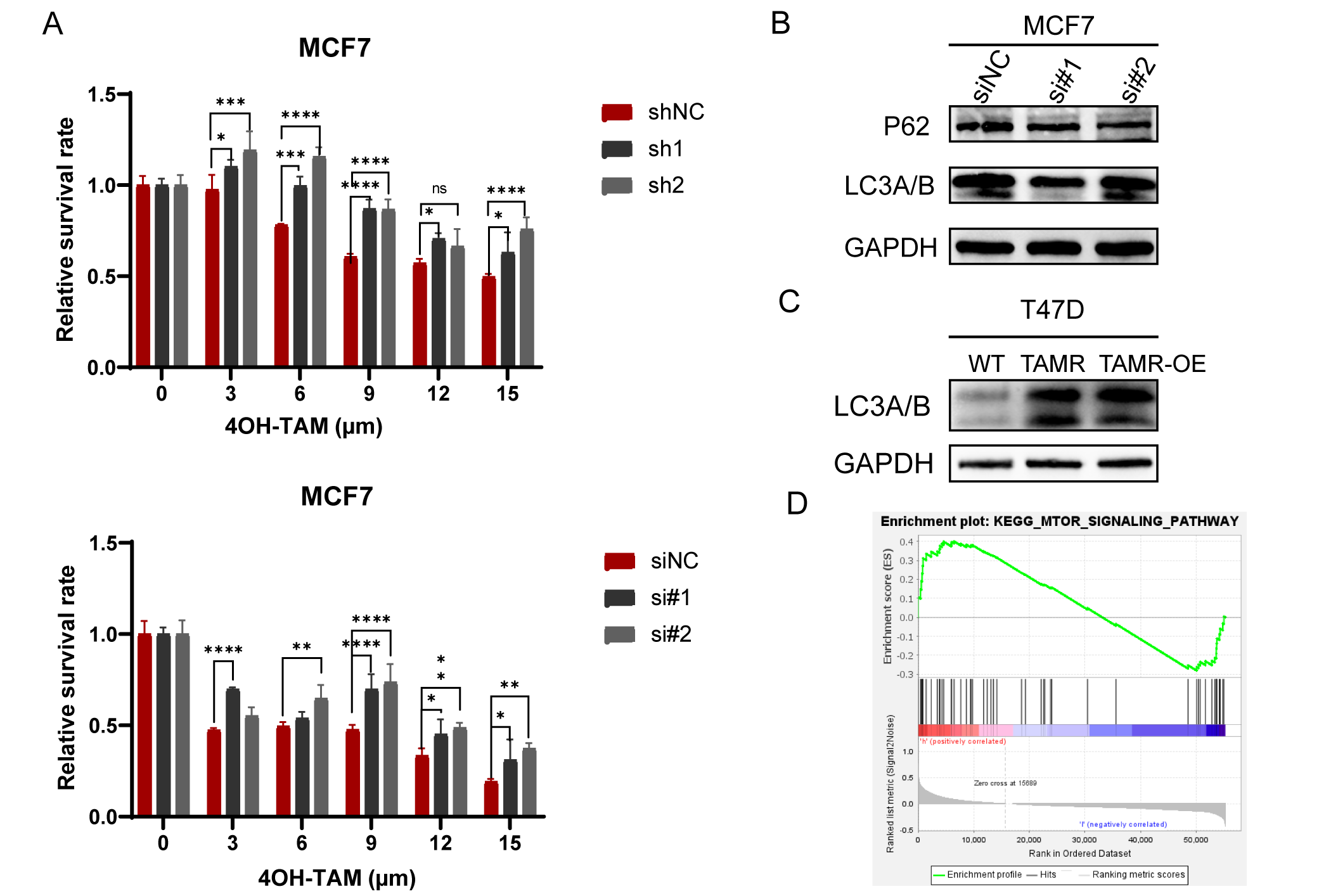

Supplement: Supplementary file 1 [file life-12-01461-s001.zip › life-1874856-supplementary/Supplement Figure/Supplement figure2-.tif]
